# Repetitive Elements May Comprise Over Two-Thirds of the Human Genome

**DOI:** 10.1371/journal.pgen.1002384

**Published:** 2011-12-01

**Authors:** A. P. Jason de Koning, Wanjun Gu, Todd A. Castoe, Mark A. Batzer, David D. Pollock

**Affiliations:** 1 Department of Biochemistry and Molecular Genetics, School of Medicine, University of Colorado, Aurora, Colorado, United States of America; 2 Department of Biological Sciences, Louisiana State University, Baton Rouge, Louisiana, United States of America; The University of North Carolina at Chapel Hill, United States of America

## Abstract

Transposable elements (TEs) are conventionally identified in eukaryotic genomes by alignment to consensus element sequences. Using this approach, about half of the human genome has been previously identified as TEs and low-complexity repeats. We recently developed a highly sensitive alternative *de novo* strategy, *P-clouds*, that instead searches for clusters of high-abundance oligonucleotides that are related in sequence space (oligo “clouds”). We show here that *P-clouds* predicts >840 Mbp of additional repetitive sequences in the human genome, thus suggesting that 66%–69% of the human genome is repetitive or repeat-derived. To investigate this remarkable difference, we conducted detailed analyses of the ability of both *P-clouds* and a commonly used conventional approach, *RepeatMasker* (*RM*), to detect different sized fragments of the highly abundant human Alu and MIR SINEs. RM can have surprisingly low sensitivity for even moderately long fragments, in contrast to *P-clouds*, which has good sensitivity down to small fragment sizes (∼25 bp). Although short fragments have a high intrinsic probability of being false positives, we performed a probabilistic annotation that reflects this fact. We further developed “element-specific” *P-clouds* (ESPs) to identify novel Alu and MIR SINE elements, and using it we identified ∼100 Mb of previously unannotated human elements. ESP estimates of new *MIR* sequences are in good agreement with RM-based predictions of the amount that RM missed. These results highlight the need for combined, probabilistic genome annotation approaches and suggest that the human genome consists of substantially more repetitive sequence than previously believed.

## Author Summary

Our study is concerned with a fundamental question about the human genome sequence: what is it made of? At present, approximately 50% of the genome sequence has unknown function or origin and is sometimes referred to as the “dark matter” of the human genome. We demonstrate here that approximately half of this uncharted territory is in fact comprised of repetitive or repeat-derived sequences, which are most likely dominated by transposable elements. These sequences are too diverged or degraded to be easily detected by alignment to known transposable element consensus sequences, but can be detected using novel *de novo* search methods that we present and evaluate here. Standard methods for detecting repetitive sequence are therefore probably missing large numbers of transposable element fragments. In one case (*MIR* elements), we predict that half of the sequence that is likely present in the genome has gone undetected. Genome-wide, we infer that a large majority of the human genome sequence (>66%–69%) is comprised of repetitive and repeat-derived DNA elements, after controlling for false positives. This estimate stands in stark contrast with previous estimates and suggests that transposable elements have played a much larger role in shaping the history and content of our genome than previously believed.

## Introduction

Eukaryotic genomes contain millions of copies of transposable elements (TE) and other repetitive sequences. Indeed, approximately half of the sequence content of typical mammalian genomes tends to be annotated as TEs and simple repeats by conventional annotation methods. By contrast, only about 5–10% of mammalian and vertebrate genome sequences comprise genes and known functional elements [1], [2], [3]. The remaining 40–45% of the genome is essentially of unknown function, and is sometimes referred to as the ‘dark matter’ of the human genome. The origins of this ‘dark matter’ fraction of the genome have presumably been obscured, in part, by extensive rearrangement and sequence divergence over deep evolutionary time. Understanding the content and origins of this huge uncharacterized component of the genome represents an important step towards completely deciphering the organization and function of the human genome sequence [4], [5], [6].

The dominant repeat annotation paradigm focuses on the identification of repeat element sequences via alignment to consensus TE sequences, as in the widely-used RepeatMasker (RM) approach [7]. Such approaches rely on well-curated libraries of known repeat family consensus sequences, which are usually provided by Repbase [8]. Thus, methods like RM can be described as not masking repeats, per se, but rather masking sequences with clear similarity to repeat consensus library sequences. Ultimately, alignment-based approaches are designed, and tuned, to conservatively mask regions that are clearly identifiable as TEs. Such approaches are therefore expected to be most effective for well-studied genomes with long histories of repeat library curation [9], [10], [11], . Even when TE databases are well-curated, however, there are plausible circumstances where such methods might be expected, *a priori*, to have poor sensitivity. Consensus sequences may not align well to old and highly diverged TE family members, for example, and alignment-based approaches may have trouble identifying short segments [17].

If half of the human genome can readily be identified as belonging to known TE families, it would seem reasonable to assume that much of the unannotated genomic dark matter may also be derived from TEs [18], even if the precise origins of such sequences are difficult to identify. TE elements have long been active in vertebrate genomes, and different families have diversified to varying degrees and at different times along the lineages leading to present-day species [19]. As a result, we expect that hundreds of millions of years of vertebrate evolution would have heavily altered substantial amounts of TE-derived sequence. Insertion, deletion, and sequence divergence would make many such elements quite difficult to identify. We postulated, however, that mutations in these ancient TEs will have produced a great deal of related but diverged sequences, and that the relations among these large clusters of sequences may make them detectable, even when individual sequences are not.

**Figure 1:**
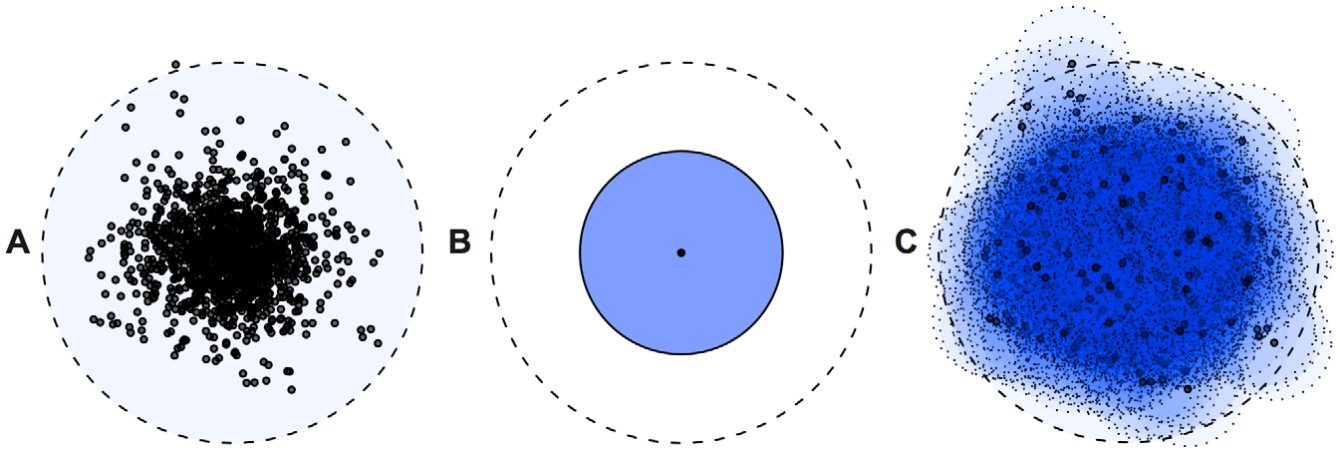
Principles of repeat identification using *P*-clouds. A) True data distribution representing divergence within a TE family from a master element sequence (center). B) Consensus sequence based search throws away information by collapsing observed data to a single sequence. C) *P-clouds* clusters related high-abundance oligos, thus providing better coverage of sequence space.

Motivated by these arguments, we developed a novel approach to identify and demarcate likely repetitive regions in large genomes [13], [20], [21]. This approach first identifies short oligos that are highly repeated (similar to some other de novo repeat-finding methods; [22]), but then groups closely-related oligos that occur, as a group, more often than predicted by chance (Figure 1). These ‘*P-clouds*’ are then used to demarcate regions of the genome that are of putatively repetitive origin. Identification of putative repeat-derived regions using *P-clouds* is far more rapid than consensus-based alignment identification of TEs, and analysis of the human genome can be accomplished on a modest desktop computer in well under a day [20].

Early *P-clouds* results on two human chromosomes indicated that a reasonably large fraction of the human genome is likely to be of repetitive origin, but not annotated by RM [20]. We thus decided to examine the entire human genome here, evaluating evidence that regions uniquely annotated by *P-clouds* do indeed represent TE-derived sequences, and addressing how such regions can be better annotated using related approaches. To enable family-specific annotation, we introduce an ‘element-specific’ *P-clouds* approach (‘ESP’), that builds oligo-based *P-clouds* from sets of all known family members for a particular repeat family. These ESP's can then be used to sensitively interrogate genomes for novel fragments belonging to that family, while carefully controlling expected false positives.

To help explain genome-wide differences in inferred repetitive content between *P-clouds* and RM, we analyze the reliability of *P-clouds* and RM methods for identifying different sizes of fragments from two large and well-known families of human SINEs: Alu and MIR. These TE families were chosen to represent extremes of expected detection sensitivity. Alu elements have undergone extensive recent expansion and are generally similar in sequence [4], whereas MIR elements underwent a more ancient expansion in mammals and their sequences therefore tend to be more divergent [23]. By constructing ESPs for both of these SINE families, we identified large numbers of new, unannotated Alu and MIR fragments, throughout the human genome. Where possible, we confirmed that the numbers of these newly identified fragments closely match predicted false-negative estimates for *RM*. Our results therefore eliminate a sizeable fraction of the previously unannotated dark-matter fraction of the human genome, and provide strong evidence that a large majority of the human genome is repetitive or repeat-derived.

## Results

### How much of the human genome is repetitive or repeat-derived?

**Figure 2:**
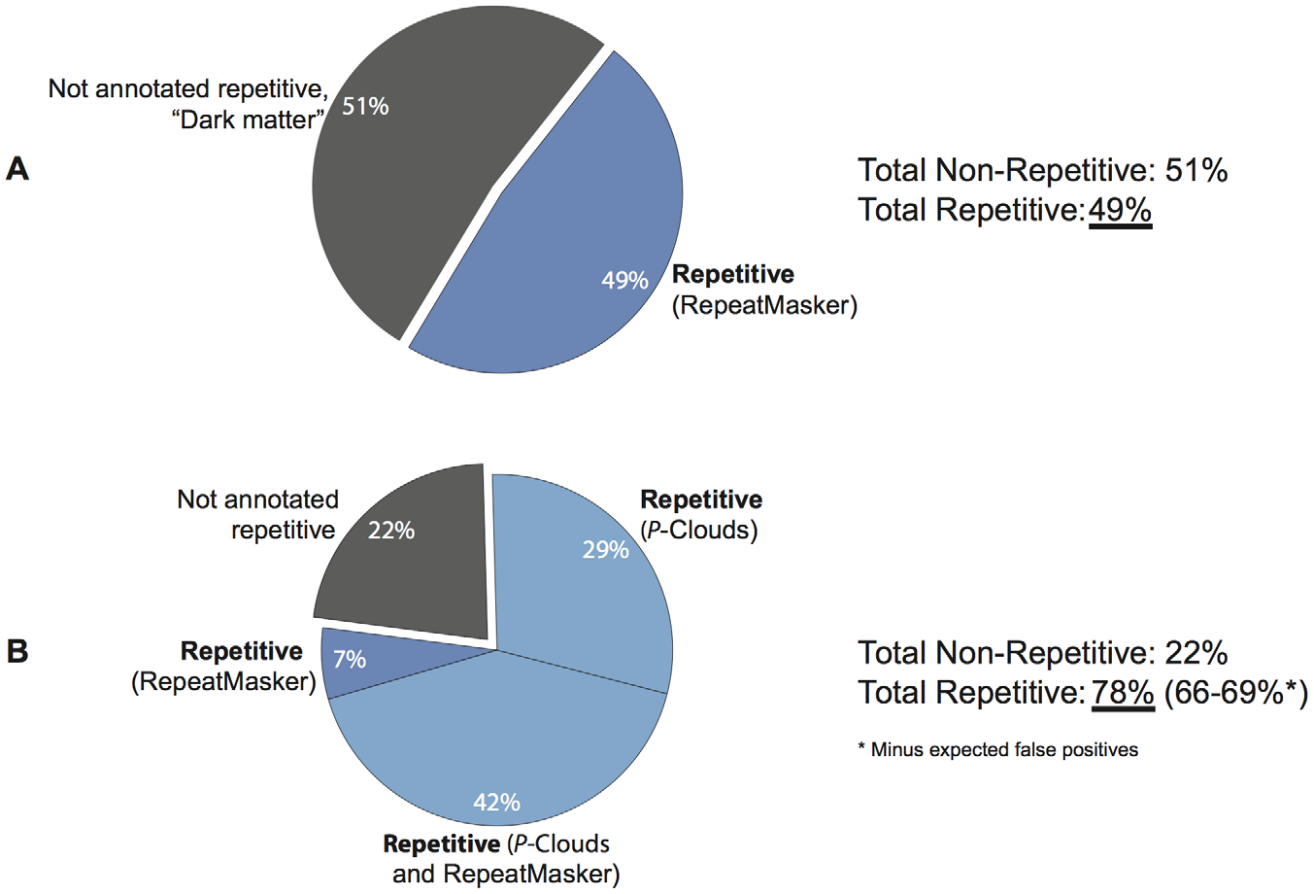
*P*-clouds and RepeatMasker annotation of the repeat structure of the human genome. Results are displayed as a percentage of the ungapped genome assembly length. A) Consensus results prior to this study indicate that <50% of the genome is repetitive (*RepeatMasker*). B) Analysis using *P-clouds* suggests more than two-thirds of the genome is repetitive or repeat-derived.

**Figure 3:**
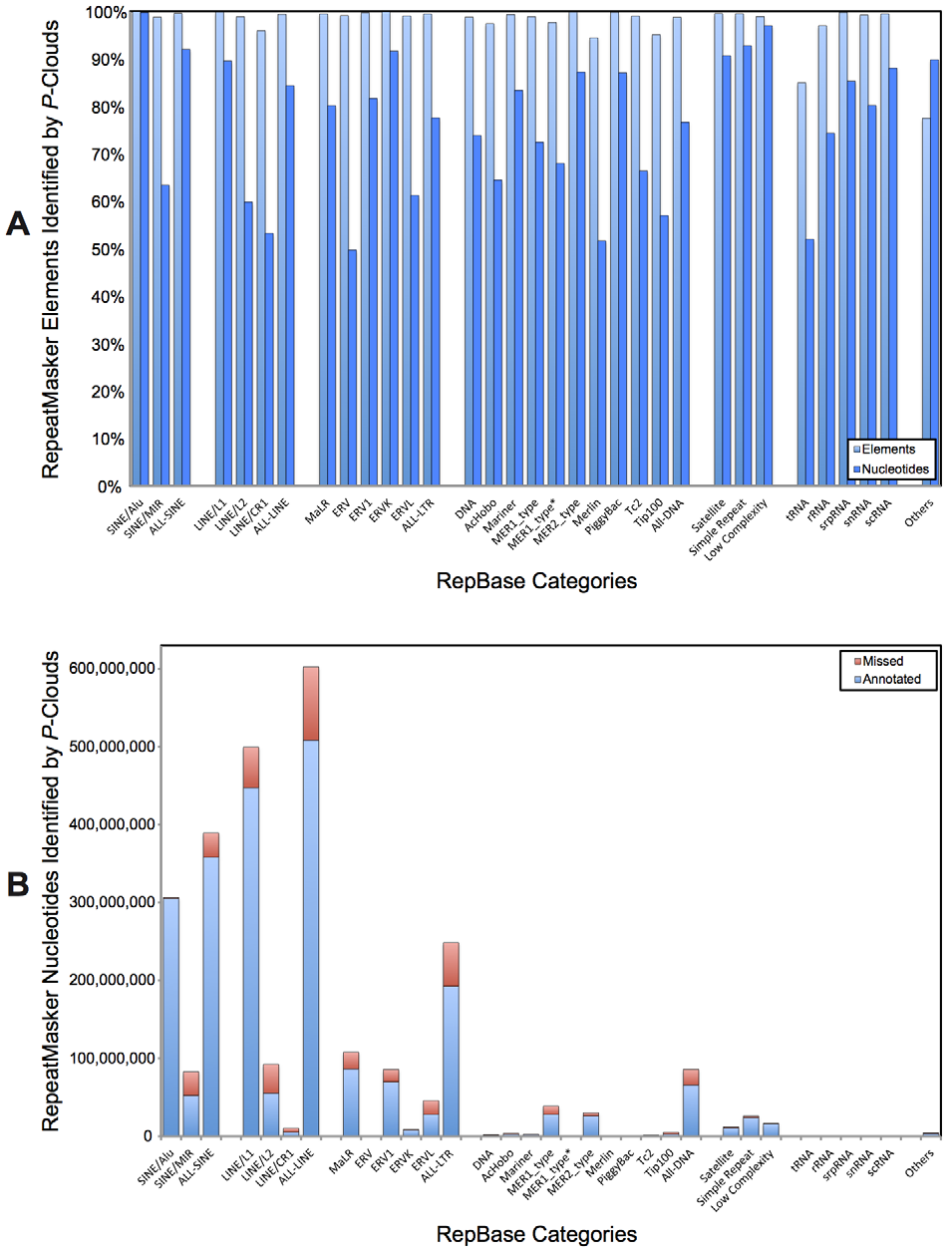
Percentage of previously-identified transposable elements annotated by *P-clouds*. A) The percentage of nucleotides and repeats for each family or repeat classification group. B) The number of nucleotides annotated or missed.

Using conservative settings, *de novo P-clouds* and RM jointly identify 78.1% of the human genome as being repetitive or repeat-derived (Figure 2), a remarkable increase of 28–33% of the genome (801–944 Mbp) over the generally accepted RM-based estimate of 45–50% [24]. *P-clouds* identifies 85.3% (1.18 Gbp) of the nucleotides in the RM annotation as being of repetitive origin, including at least some nucleotides in 95%–100% of element copies and including nearly all TE families as defined by RM (Figure 3). These results therefore suggest that *P-clouds* has good sensitivity, and is able to detect, de novo, most transposable elements that are detectable by RM using the Repbase consensus library. Not surprisingly, the distribution of novel *P-clouds* annotations is heavily biased towards short segments, although >462.7 Mbp of novel annotations (16.2% of the entire genome) were in segments of 50 bp or longer (Table S7). The primary *de novo P*-clouds annotations for the human genome are available as data tracks in the UCSC Genome Browser (provisional links are provided in Text S1); each annotated region is assigned a score based on its probability of being truly repetitive (see Methods). Overall, the average false-positive rate for P-clouds analysis from simulated non-repetitive genomes under the conditions used was estimated to be 12.6% (Table S1). This suggests that over two-thirds of the human genome is expected to be truly repetitive or repeat-derived (65.8% to 69.1%, using the average FP rate across all fragment sizes, or using the length-dependent FP predictions, respectively; Table S6).

We next considered whether repeat annotations were strongly associated with particular sequence categories such as known genes, segmental duplications, and CpG islands. For each category, we compared the frequency of the category in the whole genome to the frequencies in RM, *P-clouds*, and novel *P-clouds* annotations (Table 1; Mbp are provided in Table S10). Although all frequency differences are highly significant due to the large amounts of data, the frequencies of each category in the RM and *P-clouds* annotations are qualitatively quite similar to their frequencies in the genome as a whole. This means that the novel *P*-cloud annotated regions cannot be explained by a propensity to target non-TE regions.

**Table 1:** Overlap between genome features and repetitive regions.

Genome Feature	Fraction of Genome	Fraction of RepeatMasker annotations	Fraction of *P-clouds* annotations	Fraction of Novel *P-clouds* annotations
Known Genes (transcribed unit)	37.48%	32.47%	36.02%	41.42%
Segmental Duplications	5.22%	5.33%	5.75%	6.02%
Duplicated Regions (WSSD)	3.53%	3.16%	3.87%	4.63%
Known Genes (exons)	1.12%	0.05%	0.56%	1.29%
Simple Repeats	1.91%	3.00%	2.36%	1.06%
CpG Islands	0.74%	0.07%	0.26%	0.56%
Pseudogenes	0.19%	0.07%	0.16%	0.28%
Total Size:	2.85 Gbp	1.39 Gbp	2.02 Gbp	0.84 Gbp

Total repetitive sequence detected by either RepeatMasker or *P-clouds* was 2.23 Gbp (out of a total 2.85 Gbp sequence in the ungapped assembly).

In particular, the two segmental duplication categories (“Segmental Duplications” and “Duplicated Regions”) are only moderately enriched in the novel *P-clouds* annotations. It is expected that segmental duplications should not be enriched in *RM* annotations because segmental duplications should not affect transposable element frequencies, and in fact segmental duplications are at similar frequencies in the genome and *RM* annotations. *P-clouds*, on the other hand, is not limited to detecting TEs, and may therefore detect recent segmental duplications with many copies. The two segmental duplication categories are almost certainly strongly overlapping, as they are intended to measure the same thing. However, if we conservatively assume that they do not overlap at all, the excess enrichment of these categories in the novel *P-clouds* annotations is only 1.9%. This excess detection of segmental duplications thus accounts for at most only about 18 Mbp out of 839 Mbp of novel *P-clouds* annotations, and is not a major explanation for the novel *P-clouds* annotations overall.

Likewise, *P-clouds* might be expected to frequently annotate exons, CpG islands, and pseudogenes if gene families are highly repetitive and/or recently expanded or pseudogenized. Only a small proportion of *RM* annotations overlap with exons and CpG islands, probably due to rare incorporation of TEs or TE fragments in functional regions (*e.g.*, [25]). CpG islands are less represented in novel *P*-clouds annotations than in the overall genome, and the relatively higher proportions of exons and pseudogenes in novel *P*-clouds annotations add up to an excess of only 0.26% (2.2 Mbp). Thus, as with the segmental duplications, excess detection of genic regions is not a major explanation for the novel *P-clouds* annotations overall.

Simple repeats (*e.g.*, microsatellites) are moderate length sequences that are highly repetitive. Such repeats are generally present in the *Repbase* library, but the full spectrum of possible repeats is not represented, and only 76% of them (41.6 out of 54.4 Mbp; see Table S1) are detected by *RM*. Long and perfect simple repeats are excluded before *P-clouds* construction (see [20]), but nevertheless *P-clouds* annotations detect an additional 8.9 Mbp of simple repeats that *RM* did not detect, yielding a joint detection rate of 93%.

### Details of P-cloud annotation across TE families

**Figure 4:**
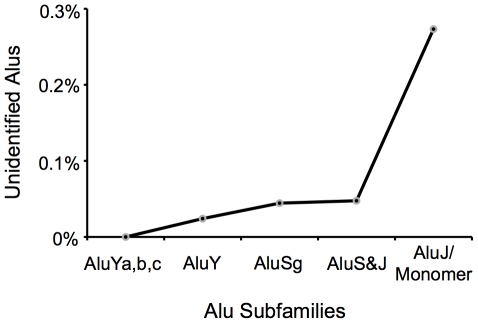
Percentage of Alu elements in different Alu subfamilies not annotated by *P-clouds* analysis. Displayed are elements for which no portion was annotated. The relative age of *Alu* subfamilies increases from left to right.

P-cloud annotation often failed to extend to the full length of a repeat element, depending on how oligo composition changed along the length of an element, but it still generally detected the presence of an element (Figure 3A). As might be expected, *P-clouds* tended to annotate nearly the full extent of element nucleotides for recently diverged (young) TE families (e.g., Alu and L1 elements), and tended to miss a larger number of nucleotides in more anciently active repeat families (e.g., MIR, CR1, and L2 elements; Figure 3B). The effect of age on detectability extends to subfamilies as well. For example, although *P-clouds* annotated at least part of 99.7% of all human Alu elements from each subfamily, the proportion of elements missed increased with the relative age of the Alu subfamilies, being lowest for the young AluY subfamily and highest for AluS, and AluJ/Alu Monomer subfamilies (Figure 4). Although the total number of elements in a family should make some difference in detectability, age was more important, and small families were still well-detected (Figure 3B). The *P*-cloud approach, like all repeat annotation approaches available, therefore shows decreased sensitivity to detect more ancient repeat elements. An important difference, however, is that this drop in sensitivity is far less severe for *P-clouds* than it is for conventional detection methods (examined below). Nevertheless, the increased difficulty of detecting old, highly-diverged repeat sequences by all methods is a reason to expect that we are likely underestimating the true proportion of repeat-derived regions, even when taking the union of *P-clouds* and *RM* results.

### Conservativeness of *P*-cloud construction

Parameter settings for P-cloud analyses were adjusted to avoid excessive false positives, which resulted in only 43% (194 million out of 451 million) of oligos that were observed more than once being included in *P*-clouds. Most oligos with fewer than ten copies in the genome were also not included in *P*-clouds, even though the observed frequency of these low copy oligos was far above chance expectations (Figure S2). This result implies that the P-cloud parameter settings used may have excluded a substantial fraction of repeat-derived oligos. Thus, although *P-clouds* is capable of discovering a large set of novel repeat-derived sequences in the human genome, estimates from *P-clouds* under the settings used are nevertheless likely to still be an underestimate of the true proportion of repeat-derived sequences.

### Ability to detect fragments of human Alu and MIR elements

If the novel P-cloud annotated regions are in fact repeat-derived, it is likely that many of them arose from TEs, since the largest fraction of previously-annotated repeat regions are TEs. This implies both that RM must miss a large number of TEs, and that the *de novo* P-cloud method is capable of finding them. To test both of these implications, we examined the performance of both methods for detecting human Alu and MIR elements – two SINE element families with different age distributions and therefore different intrinsic detectabilities. Detection performance was measured by the ability of each method to detect segments of 1,000 known elements interspersed among simulated non-repetitive genomic sequence.

**Figure 5:**
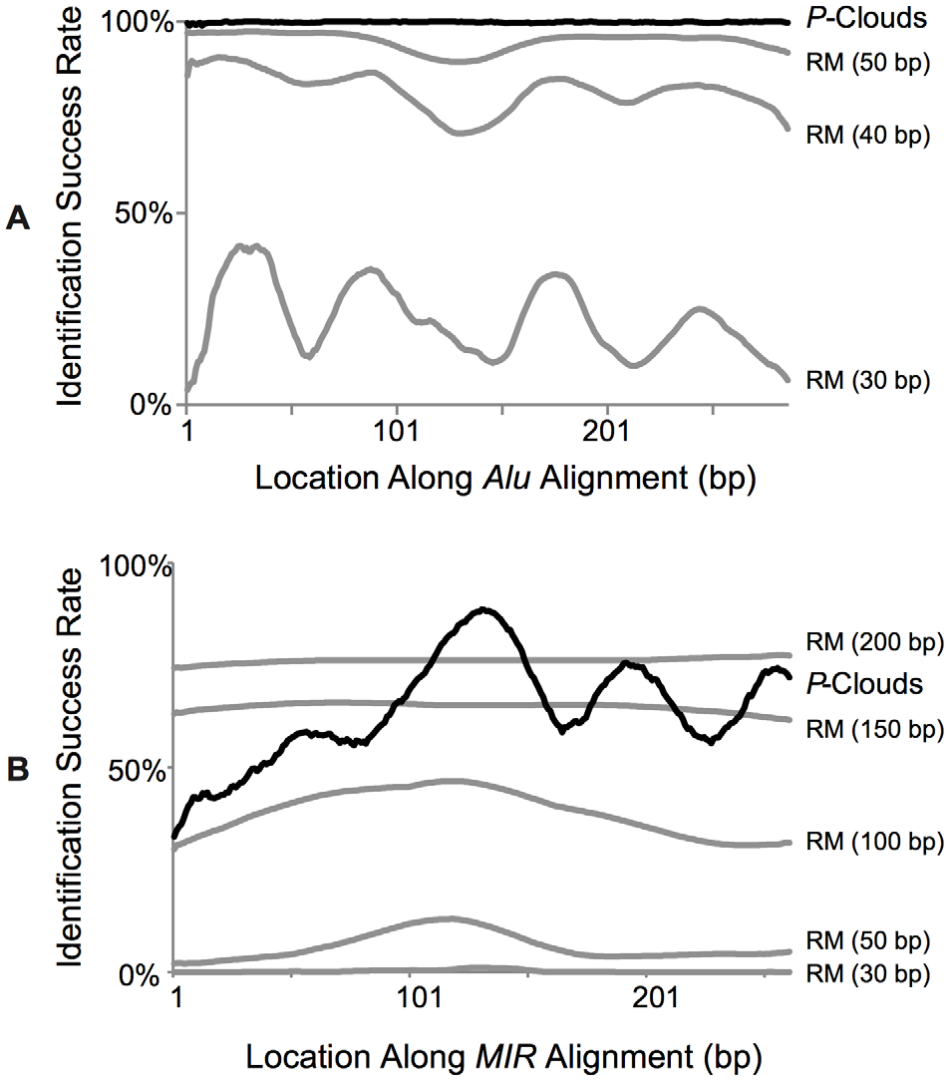
Percent detection success for fragments of known full-length *SINE* elements. A) *Alu* regions. B) *MIR* regions. Identification success is displayed as a running average of 10 bp starting positions.

Given that *Alus* are so abundant and well-studied, RM has surprisingly low sensitivity to detect Alu fragments, particularly for short (30 or 40 bp) fragments (Figure 5A). On average, RM failed to detect 77.9% of 30 bp fragments and 18% of 40 bp fragments. Detection success varied depending on what region of the full-length Alu element the fragment represented; this is presumably because some regions of Alu elements have diverged more than other regions from the consensus “master element” sequence. Even for 50 bp segments, 5.1% were missed by *RM*. In contrast, using standard settings (16-mers and a demarcation criterion of 80% coverage in 10 consecutive oligos), the *de novo* P-cloud algorithm can reliably detect TE segments as short as 24 bp, and detected over 99.8% of these small Alu fragments across different Alu regions (Figure 5A; discussed below).

In contrast to the younger and highly-abundant *Alu* SINEs, MIR elements are more difficult for both methods to identify, but are far more difficult for RM than *P-clouds*. RM could barely detect 30 and 50 bp fragments (only 6% of 50 bp fragments, on average), and detected 30–50% of 100 bp fragments, depending on the region of the element (Figure 5B). Even for 200 bp fragments, which is nearly full length for MIR elements, only 76% were detected. The P-cloud method in contrast detected an average of about 64% of 24 bp fragments across different regions of MIR elements. Detection was better for fragments in the middle of MIR elements, and worse near the beginning, a pattern also seen with RM, but not as pronounced.

These results demonstrate that RM greatly underestimates the amount of TE-derived sequences present, and therefore strongly suggest that much of the newly-annotated P-cloud regions may have arisen from TEs undetected by RM. RM missed a large proportion of fragmented TEs that it was able to positively identify when they were part of full-length segments. The problems with the consensus approach (as exemplified by RM) are much worse for the older MIR elements of all sizes, and surprisingly consequential for short Alu elements. In contrast, *P-clouds* was able to find many of the elements that RM missed, and was insensitive to TE fragmentation for fragments over 24 bp.

### Performance of element specific *P-clouds* (*ESP*s) for detecting Alu and MIR elements

To specifically identify members of TE sub-families of interest, we introduce here the concept of ‘element specific *P*-clouds’ (or *ESP*s), which build an oligo cloud from a set of *known* elements, and then annotate the genome for regions containing a high density of element-specific *P*-cloud oligos. To evaluate the effectiveness of this approach, we used *Alu*- and *MIR*-specific *P*-clouds to search for previously unannotated *Alu* and *MIR* sequences in the human genome. Specifically, we searched the portion of the human genome that is not masked by *RM*, the putatively “non-repetitive” portion of the genome.

This approach detected 749,395 putative Alu regions totaling 20,919,291 bp, and 7,518,362 putative MIR regions, totaling 227,472,397 bp. The overall false positive rates were high, based on predictions from dinucleotide simulations: it is expected that, on average, 22.17% and 65.42% of these nucleotides represented false positives, respectively. Given that short segments are most difficult for RM to identify, it is unsurprising that most of the novel TE regions detected in the RepeatMasked genome were short (Tables S8 and S9). The shortest of these fragments accounted for most of the expected false positives, while the slightly longer segments were both abundant and reliably detected. For example, for Alu, there were 4,837 new elements ≥50 bp (totaling 274,219 bp), while for MIR there were 388,264 such elements (totaling 23,202,343 bp). For these slightly longer elements, the expected false positive rates were only 0.16% for Alu, and 2.3% for MIR (summarized in Tables S4 and S5). To fully account for this great range in expected false positive rates, we performed all annotations probabilistically by assigning each predicted element a probability of being a true or false positive result based on its length (see Methods). After accounting for false positive results in this way, we estimate that there are 571,229 putative Alu elements (20,333,327 bp) and 2,249,431 putative MIR elements (78,654,070 bp) present in the *RepeatMasked* portion of the human genome (Tables S8 and S9). This corresponds to an increase in total family-specific nucleotides of 6.6% for Alu and 94.8% for MIR.

**Figure 6:**
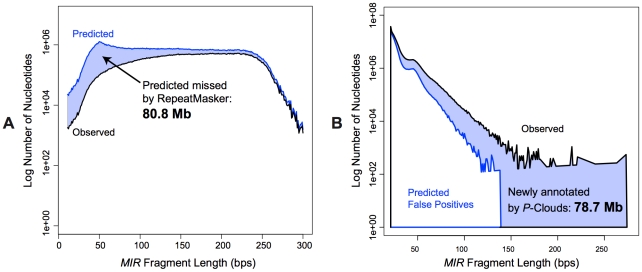
MIR element-specific *P-clouds* detect the short fragments that *RepeatMasker* cannot. A) Predicted true distribution of *MIR* fragments in the human genome, using observed *RepeatMasker* results and *RepeatMasker's* sensitivity estimates from Figure 5B. B) Novel *P*-clouds annotations on the RepeatMasked portion of the human genome, minus predicted false positives from dinucleotide simulations (see text).

The distributions of these elements are consistent with the hypothesis that there is a drop-off in element detection sensitivity for RM at smaller sizes, which *P-clouds* is far less susceptible to. To test this idea, we used the detection sensitivity of *RM* for different average fragment lengths (Figure 5B) to estimate how many *MIR* elements were probably missed by *RM* across the entire genome (Figure 6A). This analysis predicted that 80.8 Mbp of *MIR* elements were likely missed by *RM*, which corresponds quite well with the amount of novel *MIR* sequence predicted using *ESP*s after accounting for false positives (78.7 Mbp; Figure 6B). This close correspondence supports the hypothesis that *P-clouds* identified many of the short fragments that *RM* had difficulty detecting, and suggests that the difference in sensitivity between methods probably explains a great deal of the overall differences in repeat annotations (Figure 2).

### Effect of consensus library use on RepeatMasker performance

To explain, at least in part, why *RM* detected so much less repetitive sequence than *P-clouds*, we performed a detailed comparison using various element libraries for the putative MIR element fragments detected by *ESP*s (which tend to be longer than the putative Alu fragments, and therefore were more practical to work with). RM was run with standard settings against the putative MIR regions using three libraries: a) the consensus *MIR* sequence in Repbase; b) all known full-length human *MIR*s; and c) all known human *MIR* elements. Using the Repbase consensus sequence, only 37 putative *MIR* regions (3,402 bp) with a mean length of 91 bp were confirmed. In contrast, using full-length *MIR*s, 14,636 *MIR* elements (743,685 bp) were confirmed, and 40,602 novel elements (2,039,475 bp) were confirmed by *RM* using all known *MIR* elements as the reference library. Thus, when we expanded the library from the Repbase consensus sequences to all known *MIR* elements, we were able to many detect more elements, and most of them were around 50 bp (not shown). These results demonstrate that without changing any parameter settings, and only expanding the repeat reference library in a reasonable way, *RM* is capable of confirming a substantial fraction of the elements around 50 bp or longer that standard *RM* analyses missed but that *ESP*s identified. This finding thus supports the reasonableness of the *P-clouds* and *ESP*-based estimates, while again suggesting that *RM* underestimates genome repeat-content. It is notable that while these results suggest that using all known TEs (rather than consensus sequences) would increase *RM* sensitivity, this is not computationally feasible; the *MIR*-specific analyses done this way on only the *P-clouds* predicted segments were extremely time consuming.

### Validation of novel *ESP*-identified *MIR* elements using BLAST

Continuing with our focus on *MIR* elements, each novel MIR element prediction was searched against all previously-known MIR sequences using BLAST to further validate the *ESP*-identified *MIR*s. Almost all (98.4%) of the putative MIR-derived regions matched at least one known MIR element with an E-value<1.0 (i.e., with a score expected to be observed fewer than one times in a random dataset). Most (81.5%), however, had moderately high BLAST *P*-values (>0.01; calculated using standard BLAST score statistics [26]), which is presumably one reason why *RM* was unable to detect many of them. The remaining putative MIR elements (18.4% or 1,337,390 regions) had highly significant matches (*P*≤0.01) and were enriched for longer fragments compared to the rest of the hits. For example, while there were 2,067 putative MIR elements that matched at least 50 bp of known *MIR* sequences with *P*>0.01, there 5,437 such alignments with *P*≤0.01. The BLAST-matched regions tended to be shorter than the full P-cloud annotated regions, possibly because the BLAST approach was capable of identifying only more conserved core regions of the putative MIR elements (Figure 5B). The total aligned region for the most significant matches (P≤0.01) for each novel fragment was 32,818,501 base pairs, or 14.4% of the total for newly-annotated regions. In summary, these results further support the conclusion that the MIR-specific *P-clouds* approach likely identified a substantial amount of genuinely MIR-derived sequences that were previously undetected by RM.

### Relationship to other *de novo* estimates

A variety of *de novo* repeat finding methods have been proposed (*e.g.*, [20], [22], [27], [28], [29], [30], [31], [32]), and are based on a diverse set of approaches (see [33], [34] for reviews). To assess how many of our findings on overall repeat content could be attributed to unique aspects of our approach, we chose two other representative methods for a limited comparison: *RepSeek* [27] and *RepeatScout* [22], both of which make use of oligo frequencies. *RepSeek* shares an emphasis with *P-clouds* on direct *de novo* detection of individual repeated sequences in genomes, although it differs in many details from our method. In contrast, *RepeatScout* is a widely used method designed for detecting families of repeated sequences, and must be used in conjunction with a program such as *RM* to secondarily annotate individual elements in a genome.

We found that when used as a *de novo* detection method on large highly repetitive chromosomes such as found in humans, the *RepSeek* program exceeds reasonable memory requirements (also see supporting documentation for [27]). *RepSeek*, for example, was unable to complete its seed extension phase for human chromosome 1 without running out of memory (on a machine with 500 GB of RAM). Indeed, the computational challenges of performing *de novo* approximate repeat analyses on repeat-rich vertebrate genomes are substantial. This is one reason why we chose not to pursue some of the more elegant but computationally demanding aspects of the *RepSeek* approach when we implemented *P-clouds* [20]. Nevertheless, we were able to make a comparison of the methods by analyzing chromosome 22. *RM* (using *Repbase* consensus sequences; *RM-Repbase* hereafter in this section) detected 16.66 Mbp of repeated sequences out of 34.76 Mbp (*i.e.*, 47.9% repeat content) in the ungapped assembly of chromosome 22. *RepeatScout* in conjunction with *RM* (*RM-RepeatScout* hereafter in this section) detected less repeated sequence than *RM-Repbase*, identifying 12.82 Mbp. When combined with *RM-Repbase* annotations *RM-RepeatScout* yielded 18.24 Mbp of repeats (52.5% of the chromosome). This modest increase in detection is consistent with the 2% increase in repeat content annotations found by Price *et al.* [22] using *RM-RepeatScout* on human Chromosome X.

Using a minimum oligo seed length of 35 on the entire Chromosome 22, *RepSeek* detected 2.89 Mbp more than *RM-Repbase*, with no expected false positives. At these settings, however, it missed many sequences detected by *RM-Repbase*, and thus had an extremely high false negative rate of 43% (*i.e.*, poor sensitivity). It is expected that the false negative rate for repeats in the unmasked region will be higher because they are by definition harder to detect; they are probably both lower in copy number than the masked repeats, and likely more diverged and fragmented. Nevertheless, since the false positive rate is near zero, the observed false negative rate on easily detectable sequences can be used to provide a corrected joint (*RM-Repbase* plus *RepSeek*) minimum estimate of 21.74 Mbp of repeats in Chromosome 22, or 62.54%. Given the higher numerical estimates of the repeat fraction determined by *RM-Repbase* plus *RepSeek* and *RM-Repbase* plus *RM-RepeatScout*, and the expectation that *RepSeek* and *RM-RepeatScout* are highly conservative annotations as evaluated by *RM-Repbase* (which is itself highly conservative), these results are consistent with the probabilistic *P-clouds* result of 22.98 Mbp when combined with *RM-Repbase* (70.6% of chromosome 22). The *P-clouds* result had far fewer false negatives, as evaluated using *RM-Repbase*, and included repeat fragments as short as 25 bp if they appear significant, while *RepSeek* could not detect element fragments shorter than 35 bp with the most sensitive settings that could be used. We also ran *RepSeek* on Chromosome 22 after it had been repeat masked with *RM-Repbase* (as suggested by the authors). Using minimum oligo seeds of 17 bp, we found that it detected even fewer repeats in the unmasked region (2.31 Mbp) than the 2.89 Mbp in the previous analysis. The program thus appears to be even less sensitive using shorter oligos and and when the easily-detected repeats are excluded. Overall, although both *RepSeek* and *RepeatScout* (with *RM*) are therefore less accurate estimators of genomic repeat content due to the degree to which they sacrifice sensitivity for the sake of specificity, we view the compatibility of results on chromosome 22 as supportive of the concept that the human genome is significantly more repetitive than widely believed.

## Discussion

This study provides evidence for a compelling shift in our view of the content of the human genome. Multiple lines of presented evidence indicate that current estimates of overall repeat content are substantial underestimates of the full extent of the human genome's repeat landscape. Combined P-*clouds* and *RM* analysis of the human genome indicate that it consists of at least 66–69% repetitive sequence (after false-positive control), mostly from copies of transposable elements. The compatibility of results between *P-Clouds* and *RepSeek* on chromosome 22, despite the dissimilarity of these two methods, supports the reasonableness of our *P-Clouds* estimates and the general argument that the human genome contains substantially more repeats than previously estimated. This estimate challenges the widely accepted view that the human genome consists of 45–50% repetitive sequence. In light of this study, the 45–50% number is more reasonably interpreted as simply the easily-identifiable repeat fraction. Thus, an additional 16% of the entire human genome sequence that was previously of unknown origin can now be said to be repetitive or repeat-derived, and is most likely derived from transposable elements.

The human genome has the most exhaustively curated repeat library of any species, and thus should represent the best case for performance of currently popular library-dependent repeat identification approaches. Nonetheless, the P-cloud settings used here were fairly conservative, and excluded substantial numbers of repeated oligos. Furthermore, our probabilistic approach recognizes that it is inherently more difficult to detect shorter fragments. Thus, it annotates putative repeats throughout the genome with less certainty if they are short by quantifying the increased likelihood that such sequences may exist by chance.

We can explain a great deal of the difference between our *P-clouds*-based estimates of genome-wide repeat content and conventional estimates by recognizing that RM has lower sensitivity for detecting short sequences and sequences from older and more diverse TE families. Our analyses suggest that MIR element fragments, for example, which are only a maximum of ∼250 bp in length, have a 95% chance of being missed by *RM* if they are 50 bp in length, and >50% chance of being missed if 100 bp. Even for the most common elements in the human genome, the Alu family, *RM* appears to be likely to detect fewer than 50% of 30 bp fragments. On the other hand, *P-clouds* has good sensitivity even down to ∼25 bp fragments and detects numerous verifiable TE fragments that RM is not sensitive enough to find. Given a library of known TEs, element-specific *P-clouds*, or *ESP*s, can rapidly and accurately detect and annotate previously unidentified specific element copies, including short fragments.

Despite this, it appears likely that neither P-*clouds* nor RM is capable of exhaustively identifying all known repeat elements, even when combined. Any repeat detection approach will at some point find it difficult to detect TEs from families that expanded long ago, simply because the historical signal of homology following duplication is erased by mutations that have accumulated over time. The P-*clouds* and *RM* approaches are not immune to this problem, and even jointly they still probably provide underestimates of the true genomic TE content.

However, there are reasons to be optimistic that the true origins of eukaryotic genome sequences will continue to become better resolved. One reason is that as more of the genome is assigned to specific TE origins, the number of false positives in the remainder is reduced. Another reason is that as more genomes are sequenced, it is likely that many regions will be identifiable in at least one species even if they are not identifiable in other species, and this information may be translatable across genomes. Furthermore, as more genomes are compared, recent TE insertions and expansions can be removed, enhancing the detection of anciently inserted and subsequently fragmented TE sequences. Finally, we expect that the methodology for false positive prediction in P-*clouds* may be improved with further theoretical development, leading to more accurate prediction of novel elements.

Further research to more thoroughly identify and annotate eukaryotic genome repeat structure is highly motivated by the benefit that genes may be more easily predicted if the amount of genomic ‘dark matter’ is reduced by more thorough annotation. Although numerous cases of genes having co-opted regions of transposable elements are known, repetitive regions are not in general likely to extensively overlap with coding regions. As a result, our more thorough annotation of the human genome's repeat landscape eliminates almost half a gigabase of sequence from consideration when searching for novel or unannotated genes.

Finally, it is worth commenting on the applicability of approaches such as those used here to non-human genomic sequences. Because *de novo* repeat finding tools, including *P-clouds*, can annotate repeated sequences in the absence *a priori* knowledge of repeat family structure or content, they are extremely useful for analysis of novel and uncharacterized genomes. For example, the original *de novo P-clouds* method [20] was used in the analysis of genome structure in both the opossum [13] and the zebra finch [21] genome reports, and also for characterizing the repeat landscape from samples of two snake genomes [35]. We suggest that the most effective current approach is to combine a variety of existing programs. In our experience [35], the main difficulty after application of *de novo* methods is the classification, annotation, and organization of the repeated sequences (but see [36] for attempts at automated approaches). This requires the application of a variety of programs as well as expert intervention, although the ‘element-specific *P*-clouds’ approach introduced here can be utilized as part of this process to more thoroughly (and probabilistically) annotate elements belonging to particular TE families. Based on our analyses of the human genome, we expect that the uncharacterized ‘dark matter’ fraction of other vertebrate genomes is likely to be similarly reduced by analysis with sensitive repeat finding approaches such as those considered here.

## Methods

### The P-clouds method

The P-clouds method is described in detail elsewhere [20], but in brief, it uses oligo counts to create clusters of similar high-copy oligos (the “*P*-clouds”), followed by annotation of regions in a genome with a high-density of P-cloud oligos. The basic concept behind this approach is that there should be greater statistical power to detect clusters of related oligos arising from duplicated and diverged sequences than there is to detect excess copies of each oligo individually (Figure 1).

Parameters that control P-cloud construction affect the number of repeats required to initiate or “seed” a cloud (the core cutoff), and the number of repeats required to include an adjacent oligo in sequence-space in the growing P-clouds (the lower cutoff). There are also three “extension distance cutoffs”, which control (based on the most frequent oligo in the growing cloud) whether the search for adjacent oligos will extend to oligos that are one, two, or three nucleotides different. These parameters are set by a simulation procedure that aims to empirically minimize false positives while maximizing sensitivity [20]. After construction, *P*-clouds are mapped back onto the genome, and regions of high P-clouds density are annotated as potential repeat regions. The element annotation criterion requires that 80% of every ten consecutive oligos belongs to a P-cloud. A reasonable oligo length is 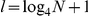, where l is the oligo word length and N is the genome or genome segment size [22]; random oligos of this length (16 for most mammals) are expected to occur in the genome less than once assuming equal nucleotide frequencies. In this study, we add a step to the original *P-clouds* protocol that assigns annotated regions a posterior probability of being repetitive that is based on the length of the putative repeat and its length-dependent false positive probability (determined by simulation as described below). The probability of being truly repetitive was calculated as 1.0 minus the probability of being a false positive; expected numbers of truly repetitive basepairs were then calculated by summing posterior probabilities across regions.

### Repeat annotation of the human genome with *P-clouds* and false positive assessment

Repetitive regions of the complete human genome assembly (UCSC genome server, 2004 May release; [24]) were annotated with *P*-clouds constructed from oligos of length 16. To perform the P-clouds analysis, parameter setting C^10^ was used, which has the following lower, core, and three extension distance cutoffs: 2, 10, 20, 200, and 2000. These settings were previously determined to represent a reasonably conservative balance between accuracy and sensitivity for complete mammalian genomes [20] (see also Table S1). The probability of false positive identification of repetitive regions was estimated by simulating a random non-repetitive genome sequence constrained to have the same dinucleotide frequencies in 1 Mbp windows as the original human genome (as described in [20]). This sequence was then analysed using the same parameter settings as for the real human genome sequence, and observed false positive rates were recorded for every repeat length that was detected in the annotation phase. These length-dependent false positive probabilities were then used to annotate the posterior probability of repetitiveness for identified regions.

### Assessment of *P-clouds* annotation overlaps and genomic distribution

Genomic regions annotated by *P-clouds* as being putatively repetitive were output to BED files and intersected with annotation tracks from the UCSC Browser [24] using BEDTools [37]. The genomic features examined for enrichment with *P-clouds* annotations were: known genes (transcribed regions and exons), pseudogenes (“pseudoYale”), simple repeats detected by Tandem Repeats Finder [38] (“simpleRepeats”, which includes microsatellites), CpG island annotations (“cpgIslandExt”), segmental duplications (both >1000 bp in non-RepeatMasked sequence, “genomicSuperDups”, and from the whole genome shotgun sequence annotations, WSSD, “celeraDupPositive”), and *RM* annotations (“rmsk”). The fraction of the genome occupied by each sequence or annotation feature was determined by merging any overlapping features on the assembly coordinates, and then dividing the total merged feature length by the length of the ungapped human genome assembly or ungapped annotation feature. The ungapped length of the human genome was used throughout this study.

### P-clouds and RepeatMasker detection capability for fragments of known elements

To assess and compare the sensitivity of the P-clouds and RM methods for known repeat elements, we evaluated performance using human SINE element families Alu and MIR as case studies.

For each family, 1,000 full-length human elements (≥286 bp for Alu, and ≥260 bp for MIR) were randomly chosen from the UCSC genome browser's *RM* annotation track and were aligned; the genomic location and classification of these elements are listed in Tables S2 and S3. For a range of fragment sizes, every possible fragment was extracted to test the ability of RM and *P-clouds* to detect it. Based on preliminary analyses of what sizes were difficult to detect, Alu fragment sizes of 30, 40 and 50 bp were used, while fragment sizes of 30, 50, 80, 100, 150, and 200 bp were used for MIR.

To analyze detection sensitivity, repeat element fragments were concatenated with randomly chosen nucleotide sequences from the non-repetitive simulated genome (above), to create an artificial genome having 10% repeat elements that were separated by equal amounts of simulated sequence. For RM analyses, the default settings and Repbase consensus sequences (version 12.05) [8] were used. As in the other *de novo P*-clouds analyses, P-cloud settings used the C^10^ parameter combination and the standard 80% annotation criterion [20]. The detection sensitivity of each method was measured as the number of repeat element fragments that were identified (expressed as a percentage of elements correctly identified).

To predict how many repeat-element fragments might have been missed by RM (expressed in basepairs), the number of detected fragments of each size was divided by the fragment's size-specific *RM* detection sensitivity determined above, thus approximating the actual number of repetitive basepairs likely to be present in the genome. For fragments sizes not evaluated in the fragment detection analysis, the size-specific detection sensitivity was estimated by spline-based interpolation (Figure S1). The predicted number of total repetitive basepairs minus the observed number was then used as an estimator of the total number of family-specific nucleotides that were likely missed by RM.

### Element-specific *P-clouds* (*ESP*s) for specific annotation of novel Alu and MIR elements

Standard P-clouds analysis begins by computing oligo clouds from the entire genome of interest [20], which facilitates a comprehensive but non-specific annotation of all classes of repeated sequences along with their diverged copies. To enable specific annotation of particular transposable element families, we introduce here an alternative approach called ‘element specific *P*-clouds’ or *ESP*. In *ESP* analysis, the initial P-cloud construction phase is performed using all known sequences from a particular TE family, rather than from the entire genome of interest. Expected false positives assessments and probabilistic genomic annotation are then performed identically for *ESP* analysis as for standard *P-clouds*, yielding a comprehensive annotation of TE family members including diverged copies. As with standard P-clouds analysis, it is important to adjust P-cloud construction parameters to empirically control false positives.

Two element-specific *P*-clouds were built – one for Alu and another for MIR elements. To allow cross-validation of the sensitivity measurements, element-specific *P-clouds* did not include the 1,000 randomly-chosen elements in the test set described above. Sensitivity of detection using *ESP*s was assessed by estimating attained true positives in the 1,000 aligned *Alu* and *MIR* elements from above, while false positive rates were estimated based on annotation of the simulated non-repetitive genome (as described in the previous section).

Due to the different evolutionary histories of these two families, different parameter settings were used to maintain reasonable false positive rates, keeping the average false positive rate below 24.1% for Alus (2, 10, 20, 200 and 2000) and 65.4% for MIRs (1,2,4,40,40). Although these overall FP rates appear high, they drop off rapidly as a function of increasing element length (summarized in Tables S4 and S5). Furthermore, due to our probabilistic approach, short annotations with a high intrinsic likelihood of representing FPs are annotated with a posterior probability that reflects this likelihood (as determined empirically, above). This approach therefore is expected to have high power to detect shorter repeat-derived sequences, while reasonably reflecting the high probability of FPs associated with detecting short genomic features that is intrinsic to any method.

### Validation of ESP predictions

To verify the identity of as many P-cloud-annotated MIR and Alu elements as possible, RM was run on the putative elements under standard settings except that rather than using the repeat library, all previously identified human elements in each TE family (Alu or MIR) were treated as the consensus library for identifying further repeat elements. This approach was used to search for matches to the library of putative, newly identified segments from the *ESP* analyses. The motivation here is that diverged copies of known TEs might align well to the putative TE fragments in cases where the consensus sequence does not. We also repeated the same comparisons using BLAST [39] because RM enforces fairly stringent cutoff values on its sequence similarity search strategy that may be overly strict. For each P-cloud-annotated TE, the BLAST score and alignment were recorded for the best hit against RM-annotated TEs.

### Running *RepSeek*

When run on the complete human Chromosome 22 sequence, *RepSeek* was run with a minimum seed size of 35 bp (*Lmin* = 35), as shorter lengths led to memory usage being exceeded, even on a machine with 500 GB RAM. At these settings, *RepSeek*'s seed-level score statistics suggest that all annotated repeats will be highly significant (*P*<0.001 with *Lmin* = 27). In their documentation, the authors suggest running RepSeek after first removing the known repeats, so we also ran *RepSeek* on the repeat-masked human Chromosome 22 (using *RM* and *Repbase*), which we were able to do with a minimum seed size of 17, as suggested by the “repeat level score statistic” from *RepSeek*. For this oligo length, the results also have to be filtered by the repeat score statistics to make sure that they are significant.

## Supporting Information

Figure S1 Interpolation of percent *MIR* fragments successfully identified using spline regression.
(PDF)

Figure S2 The relation between copy number and number of oligos. The number of 16-mer oligos not included in *P-clouds* (circles, solid line) and the number expected based on Poisson expectation (squares, dashed line) are shown. The data shown is for a *P*-clouds analysis with parameter setting C^10^.
(PDF)

Table S1 *P*-clouds parameter settings and estimated false positive rates for complete mammalian genomes [20].
(XLSX)

Table S2 The genomic location and classification of the randomly selected 1,000 human Alu elements.
(XLSX)

Table S3 The genomic location and classification of the randomly selected 1,000 human MIR elements.
(XLSX)

Table S4 Summary of false positive estimates for *Alu ESP*s.
(XLSX)

Table S5 Summary of false positive estimates for *MIR ESP*s.
(XLSX)

Table S6 Summary of *de novo P-clouds* analysis of the entire human genome, with length-dependent false positive estimates and expected true positives.
(XLSX)

Table S7 Summary of the novel *de novo P-clouds* repeat predictions for the human genome (any overlapping *RepeatMasker* results subtracted), with length-dependent false positive estimates and expected true positives.
(XLSX)

Table S8 Details of *Alu ESP* analysis on the *RepeatMasked* portion of the human genome (with false positive predictions and expected true positives).
(XLSX)

Table S9 Details of *MIR ESP* analysis on the *RepeatMasked* portion of the human genome (with false positive predictions and expected true positives).
(XLSX)

Table S10 Overlap between genome features and repetitive regions. This is the same data as in Table 1, but in Mbp rather than percent of the category. These numbers are not adjusted for estimates of false positives in the *P-clouds* annotations.
(DOCX)

Text S1 Provisional URLs for UCSC genome browser tracks containing the *de novo* and element specific (for *Alu* and *MIR*) *P-clouds* annotations of repetitive regions in the human genome.
(DOCX)
