# Supplementary figures and images for: Repetitive Elements May Comprise Over Two-Thirds of the Human Genome
Source: PLoS Genet. 2011 Dec 1;7(12):e1002384. doi: 10.1371/journal.pgen.1002384 (PMC3228813; doi:10.1371/journal.pgen.1002384)

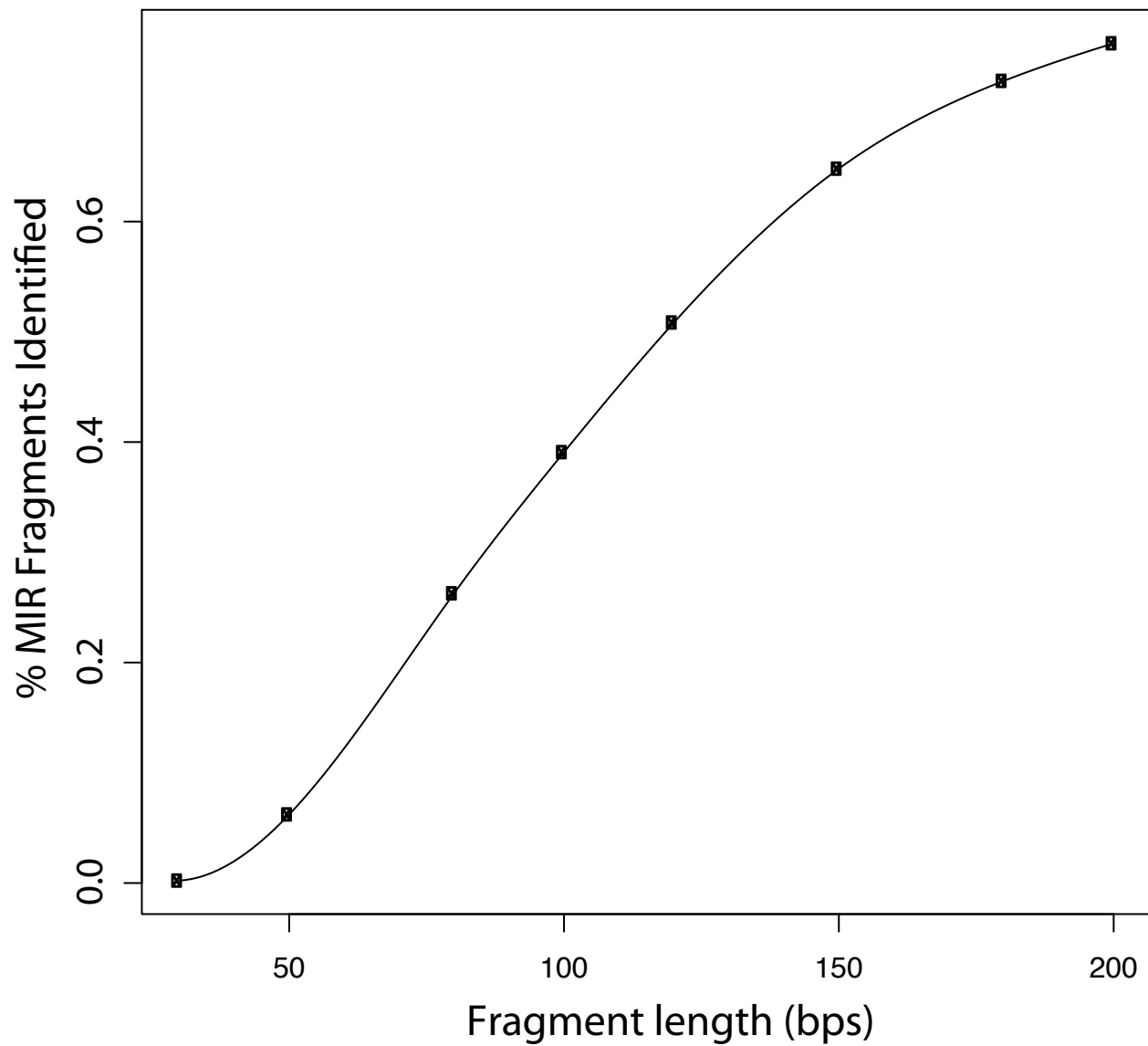

Figure S1

Supplement: Figure S1 — Interpolation of percent MIR fragments successfully identified using spline regression. (PDF) [file pgen.1002384.s001.pdf]

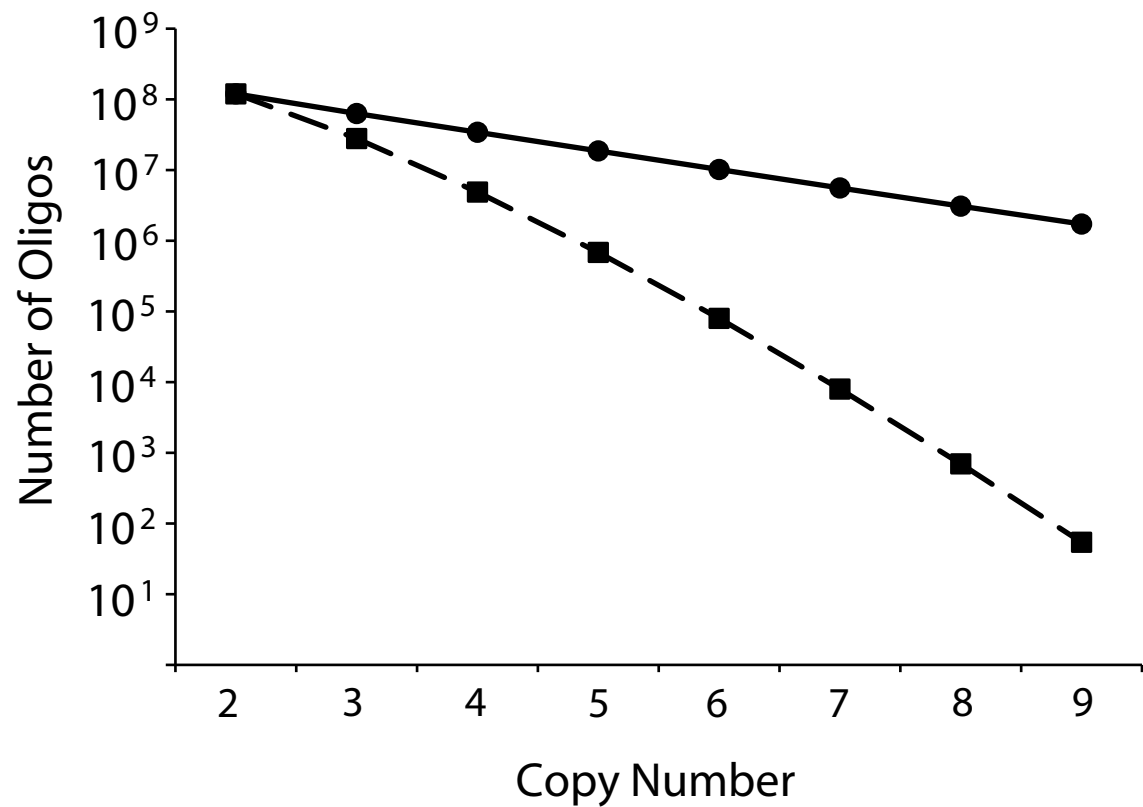

Figure S2

Supplement: Figure S2 — The relation between copy number and number of oligos. The number of 16-mer oligos not included in P-clouds (circles, solid line) and the number expected based on Poisson expectation (squares, dashed line) are shown. The data shown is for a P-clouds analysis with parameter setting C10. (PDF) [file pgen.1002384.s002.pdf]
