# Supplementary material for: Repetitive Elements May Comprise Over Two-Thirds of the Human Genome
Source: PLoS Genet. 2011 Dec 1;7(12):e1002384. doi: 10.1371/journal.pgen.1002384 (PMC3228813; doi:10.1371/journal.pgen.1002384)
Supplement: Table S10 — Overlap between genome features and repetitive regions. This is the same data as in Table 1, but in Mbp rather than percent of the category. These numbers are not adjusted for estimates of false positives in the P-clouds annotations. (DOCX) [file pgen.1002384.s012.docx]

Table S10. Overlap between genome features and repetitive regions. This is the same data as in Table 1, but in Mbp rather than percent of the category. These numbers are not adjusted for estimates of false positives in the *P-clouds* annotations.

| Genome Feature | Mbp of Genome | Mbp of RepeatMasker annotations | Mbp of  *P-clouds* annotations | Mbp of Novel *P-clouds* annotations |
| --- | --- | --- | --- | --- |
| Known Genes  (transcribed unit) | 1,068.6 | 450.4 | 728.5 | 347.5 |
| Segmental Duplications | 148.9 | 73.9 | 116.3 | 50.5 |
| Duplicated Regions (WSSD) | 100.8 | 43.8 | 78.2 | 38.9 |
| Known Genes (exons) | 31.8 | 0.7 | 11.4 | 10.9 |
| Simple Repeats | 54.4 | 41.6 | 47.8 | 8.9 |
| CpG Islands | 21.0 | 1.0 | 5.3 | 4.7 |
| Pseudogenes | 5.5 | 0.9 | 3.2 | 2.3 |
| Total Size: | **2,851 Mbp** | **1,387 Mbp** | **2,022 Mbp** | **839 Mbp** |
