# Supplementary material for: Repetitive Elements May Comprise Over Two-Thirds of the Human Genome
Source: PLoS Genet. 2011 Dec 1;7(12):e1002384. doi: 10.1371/journal.pgen.1002384 (PMC3228813; doi:10.1371/journal.pgen.1002384)
Supplement: Text S1 — Provisional URLs for UCSC genome browser tracks containing the de novo and element specific (for Alu and MIR) P-clouds annotations of repetitive regions in the human genome. (DOCX) [file pgen.1002384.s013.docx]

**Text S1.** Provisional URLs for UCSC genome browser tracks containing the *de novo* and element specific (for *Alu* and *MIR*) *P-clouds* annotations of repetitive regions in the human genome.

**1) *P-clouds de novo* track:**

<http://genome.ucsc.edu/cgi-bin/hgTracks?db=hg17&position=chr1&hgt.customText=http://jasondk.org/Pclouds/deNovoPClouds_humanAll.bb.bed>

**2) *P-clouds ESP* track (*Alu*s):**

<http://genome.ucsc.edu/cgi-bin/hgTracks?db=hg17&position=chr1&hgt.customText=http://jasondk.org/Pclouds/PutativeAlu_withFP.bb.bed>

**3) *P-clouds ESP* track (*MIR*s):** [http://genome.ucsc.edu/cgi-bin/hgTracks?db=*hg17*&position=*chr1*&hgt.customText=*http://jasondk.org/Pclouds/PutativeMIR_withFP.bb.bed*](http://genome.ucsc.edu/cgi-bin/hgTracks?db=hg17&position=chr1&hgt.customText=http://jasondk.org/Pclouds/PutativeMIR_withFP.bb.bed)
